# Molecular Analysis of Spring Viraemia of Carp Virus in China: A Fatal Aquatic Viral Disease that Might Spread in East Asian

**DOI:** 10.1371/journal.pone.0006337

**Published:** 2009-07-22

**Authors:** Nian Zhi Zhang, Li Feng Zhang, Yi Nan Jiang, Ting Zhang, Chun Xia

**Affiliations:** 1 Department of Microbiology and Immunology, College of Veterinary Medicine, China Agricultural University, Beijing, People's Republic of China; 2 Beijing Exit & Entry Inspection and Quarantine Bureau, Beijing, People's Republic of China; 3 The Key Laboratory for Preventive Veterinary Medicine of Ministry of Agriculture of China, Beijing, People's Republic of China; Institute of Infectious Disease and Molecular Medicine, South Africa

## Abstract

Spring viraemia of carp (SVC) is a fatal viral disease for cyprinid fish, which is caused by spring viraemia of carp virus (SVCV). To date, no SVC outbreak has been reported in China. Between 1998 and 2002, outbreaks of SVC were reported in ornamental and wild fish in Europe and America, imported from multiple sources including China. Based on phylogenetic analysis, the viral strain isolated from America was shown to be originated from Asia. These outbreaks not only resulted in huge economic losses, but also raise an interesting question as to whether SVCV really exists in China and if so, is it responsible for SVC outbreaks? From 2002 to 2006, we screened 6700 samples from ornamental fish farms using the cell culture method of the Office International des Epizooties (OIE), and further verified the presence of SVCV by ELISA and real-time quantitative RT-PCR. Two infected samples were found and the complete genome of SVCV was sequenced from one of the isolates, termed SVCV-C1. Several unique hallmarks of SVCV-C1 were identified, including six amino acid (KSLANA) insertion in the viral RNA-dependent RNA polymerase (L) protein and ten nucleotide insertion in the region between glycoprotein (G) and L genes in European SVCV strains. Phylogenetic tree analysis of the full-length G protein of selected SVCV isolates from the United Kingdom and United States revealed that G proteins could be classified into Ia and Id sub genogroups. The Ia sub genogroup can be further divided into newly defined sub genogroups Ia-A and Ia-B. The isolates derived from the United States and China including the SVCV-C1 belongs to in the Ia-A sub genogroup. The SVCV-C1 G protein shares more than 99% homology with the G proteins of the SVCV strains from England and the United States, making it difficult to compare their pathogenicity. Comparison of the predicted three-dimensional structure based on the published G protein sequences from five SVCV strains revealed that the main differences were in the loops of the pleckstrin homology domains. Since SVCV is highly pathogenic, we speculate that SVC may therefore pose a serious threat to farmed cyprinid fish in China.

## Introduction

Spring viraemia of carp virus (SVCV) is classified as a member of the family *Rhabdoviridae*, belonging to the genus *Vesiculovirus* [1]–[4]. The genome of SVCV is a linear single-stranded negative RNA [5], [6]. Spring viremia of carp (SVC) caused by SVCV is an acute hemorrhagic infectious disease affecting cyprinids, especially common carp (*Cyprinus carpio*) [2], [3], [7], [8]. This high mortality disease is spread in Europe, including Russia [9], America [10], [11] and part of Asia [12]. SVC has been registered in the list of contagious diseases notifiable to the Office International des Epizooties (OIE) because of its significant risk and harmfulness (http://www.oie.int/eng/normes/fcode/en_chapitre_2.1.4.htm). It is also recognized as one of class I diseases by Animal epidemic prevention law of the people's republic of China (http://www.agri.gov.cn/blgg/t20081223_1194404.htm). This means that all farmed fish in a SVC outbreak area have to be killed to prevent further spread of this virus in China.

The genome of SVCV encodes 5 structural proteins: nucleoprotein (N), phosphoprotein (P), matrix protein (M), glycoprotein (G), and viral RNA-dependent RNA polymerase (L) in the order 3′-N-P-M-G-L-5′ [2], [5], [12]. The N protein interacts with the viral RNA to form the helical structure of the nucleocapsid. The P protein associates with the L and N proteins to form the rhabdovirus nucleocapsid which is required for transcription [13]. The bullet-shaped capsid of SVCV virion consists of the M protein, which also takes part in virus assembly and budding [2], [14], [15]. The L protein interacts with the P and N proteins to achieve the transcription and replication of the virus [16]. The G protein forms trimeric peplomers or spikes on the virus surface that bind to cellular receptors, which trigger viral endocytosis [2]. It also carries neutralizing epitopes and is a potential target for DNA vaccines. So far, G proteins act as the most important antigen to determine the serological properties of rhabdoviruses [2], [4], [6]. Based on the 550 nucleotide region of G genes, the SVCV isolates can be classified into four sub genogroups: Ia, Ib, Ic, and Id [10]. The viruses in sub genogroup Ia were isolated from England and the United States. Genogroups Ib and Ic contain viruses isolated from Moldova [17], Ukraine [10], and Russia [18], respectively; and sub genogroup Id consists of viral stains isolated from in the UK [19], [20]. SVCV can infect many species of fishes. For example, natural infections were observed in common carp (*Cyprinus carpio carpio*) [3], grass carp (*Ctenopharyngodon idellus*) [21], crucian carp (*Carassius auratus*) [7], zebrafish (*Danio rerio*) and so on [22], [23]. Outbreaks of this disease are influenced by several factors, including geographic location of the pond, the age of the fishes (one month to one year old) and the water temperature [24]. The SVC usually occurs in the spring, and the incidence and mortality rate of fish can reach up to 90% when the water temperature is between 10∼17°C [8]. Studies in carp have shown that few adult fish were infected when the temperature was above 17°C, but juveniles could be infected even at 22–23°C [24]. In general, transmission of SVCV is horizontal, and the major biological vectors are the carp louse (*Argulus foliaceus*) and leeches (*Piscicola Geometra*) [25]. The latent infected carries fish and survivors may serve as the natural carriers of SVCV, causing outbreaks of SVC in the early spring [26].

SVCV was firstly isolated from infectious dropsy of carp (IDC) by Fijian et al. [27], subsequently by Ahne et al. [28]. The later, SVCV (10/3), was used as a reference and in 1998, it was given as a gift to China for research purpose by the international reference laboratory. In the last few years, outbreaks of SVC were reported in ornamental and wild fish in America and Europe [10], [11], which were imported from multiple sources including China. Specifically, based on phylogenetic data, the five viruses isolated in America were referred as “Asian strain”, since their G genes were distinct from the European reference strains [12]. The present work describes the screening of more than 200 samples collected from ornamental fish farms in 2005 using valid cultural methods of OIE, ELISA and real-time quantitative RT-PCR. The complete genome was sequenced of one isolate, strain SVCV-C1, revealing a six amino acid insertion occurred in the L protein of SVCV-C1. Viral epizooties were also studied.Based on our results, we speculate that SVC may pose a serious threat to cyprinid fish in East Asian.

## Results

### Isolation of SVCV-C1 Strain

SVCV-C1 strain was isolated from one of the five fish collected during surveillance tests in 2005. Eithelioma papulosum cyprinid (EPC) and grass carp ovary (CO) cells were inoculated with ten fold dilutions of the samples. Cytopathic effect (CPE) was developed with CPE observed in the 10^−1^∼10^−3^ dilutions for CO cells, and in the 10^−1^∼10^−2^ dilutions for EPC cells (Table 1). Subculturing SVCV also led to CPE. ELISA experiments confirmed that the isolated virus, termed SVCV-C1 belong to SVCV family (Table 1). Forty days after challenge with viruses prepared from cultured cells, the SVCV viruses could be detected in the tissues of infected fish, including gill, brain, kidney, intestine, liver, spleen, muscle, gallbladder and pancreas, and the water samples.

**Table 1:** Identification of SVCV strains using cell culture and ELISA methods.

Samples	ELISA	CPE on cell lines	
		CO	EPC
SVCV 10/3	1.10–1.21	10^−1^–10^−5^	10^−1^–10^−5^
SVCV-C1	1.87–1.19	10^−1^–10^−3^	10^−1^–10^−2^
Control	0.06–0.07	–	–

### Molecular analysis

**Figure 1:**
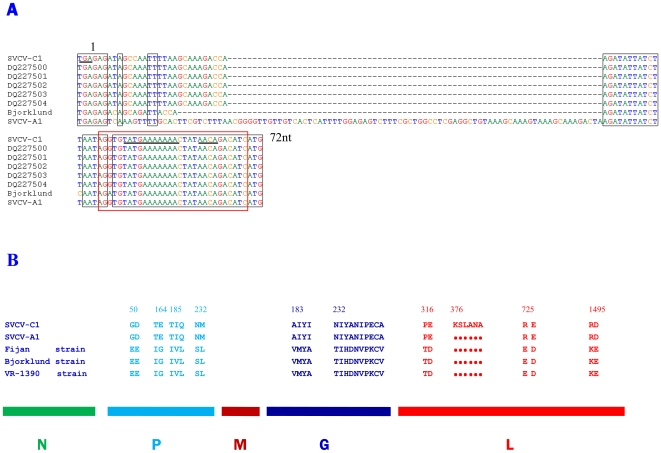
Molecular analysis of the SVCV-C1 genome. A, alignment of the nucleotide sequences in the G/L gene junction of the SVCV-C1 isolate, European reference viruses (Bjorklund) and US isolates. The stop codon of G gene and start codon of L gene are in bold. The dashes (–) indicate gaps not seen in isolate SVCV-A1. The conservative polyadenylation signal TATG[A]7 and transcription initiation sequence AACAG are underlined. B, significant diversity of protein sequences in the whole genomes of five SVCV strains. SVCV-C1 and SVCV-A1 were isolated from Asian strain and Fijan strain; Bjorklund strain and VR-1390 strain were isolated from European (comment: the three SVCV reference full length sequences used in the comparison were simply submitted by different researchers for the same virus). The different amino acids in proteins P, G and L were labeled above SVCV-C1. Six amino acids (KSLANA) inserted in the L protein of SVCV-C1 strain and dots represent deletion in other strains. The accession numbers used for analysis are: SVCV-C1, EU177782; SVCV-A1, DQ097384; Fijan strain, AJ318079; Bjorklund strain, NC_002803; VR-1390 strain, U18101; five US isolates, DQ227500, DQ227501, DQ227502, DQ227503, DQ227504.

The genomic sequence of SVCV-C1 was deposited in GeneBank under accession number EU177782 (Fig. S1). The complete genome of SVCV-C1 contains 11,047 nucleotides (nt), with 43% GC content. There are five predicted open reading frames (ORF) in the SVCV-C1 genome with a 3′–5′ direction encoding the N, P, M, G and L proteins. The first ORF is located between nt 70–1326 and encodes the nucleoprotein of 418 aa with a predicted molecular mass of 47 kDa. The second ORF (nt 1407–2336) encodes the P of 309aa with a molecular mass of 36 kDa. The ORF located between nt 2376 and 3047 encoding M protein of 223 aa with a predicted molecular mass of 26 kDa. A highly conserved PPXY motif of M protein at 17–20 aa is apparent [12]. The fourth ORF (nt 3094 to 4623) encodes the G protein of 509 aa with a calculated molecular mass of 57 kDa. There are five possible glycosylation sites in the G protein (28–31, 181–184, 338–341, 372–375, 369–372 aa). The region between nt 4696 and 11001 encodes the L protein. The putative L protein of SVCV-C1 consists of 2,111 amino acid and is larger than the L proteins from other known strains due to an insertion. The highly conserved catalytic domain is located between amino acids 569 and 786 in L protein of SVCV-C1. A conserved polyadenylation signal TATG[A]_7_ (antigenome nucleotide sequences) is seen between the 5 ORFs and the transcription initiation sequence AACAG which is identical to the corresponding sequence of vesicular stomatitis virus (VSV) is also present [5]. The putative leader region of 69 nucleotides is at the non-translated 3′ terminus, and the first 19 nt are regarded as the promoter. The transcription start signal (AACAG) of the N gene is located between nt 60 and 64. The sequence after nt 11,001 is the 5′ untranslated region and contains the transcription termination signal (TATG[A]_7_) of L gene. This region contains a sequence motif which is complementary to the leader region, a common feature of single negative stranded RNA viruses [5]. In addition to the conserved features, variations were noticeable in the junction region between G and L genes (Fig. 1A) which shares significant similarity with the corresponding region in the US isolates (DQ227500, DQ227501, DQ227502, DQ227503, DQ227504) [10]. This region is 10 nucleotides longer than that in the European reference strains but a 74 nucleotides shorter than that in the Chinese SVCV-A1 strain (SVCV-A1 was removed from GenBank by submitter, the original accession number was DQ097384).

The entire genome sequences of SVCV strains released in the GenBank were analysed. Discrepancies were observed between the Asian strains (SVCV-C1, EU177782; SVCV-A1, DQ097384) and three reference isolates (Fijan strain, AJ318079; Bjorklund strain, NC_002803; VR-1390 strain, U18101; the three SVCV reference full length sequences used in the comparison were simply submitted by different researchers for the same virus). A significant diversity of amino acid sequences was noticeable (Fig. 1B). Majority of amino acid substitutions were founded in P, G and L proteins. Further molecular analysis of predicted amino acid sequences of SVCV strains revealed an insertion of six amino acids (KSLANA) in L protein between 3376–3381 aa regions, which was a unique hallmark for SVCV-C1. The insertion was not in the highly conserved catalytic domain of L protein, but in the I domain which is one of the six conserved domains in L proteins of *Rhabdoiridae* [16]. Interestingly, in the P proteins, the amino acid substitutions occurred frequently than other four proteins and showed a wide distributed in the region 50 to 232 aa (shown a part of data). In addition, differences were seen in two regions of G protein (183–186 aa, 232–241 aa), the latter was most notable (Fig. 1B). All of these observations not only confirmed distinct features between Asian strains and European reference strains, but also revealed the limited diversity among the Asian strains.

### Analysis of the Three-dimensional Structure of G Proteins

**Figure 2:**
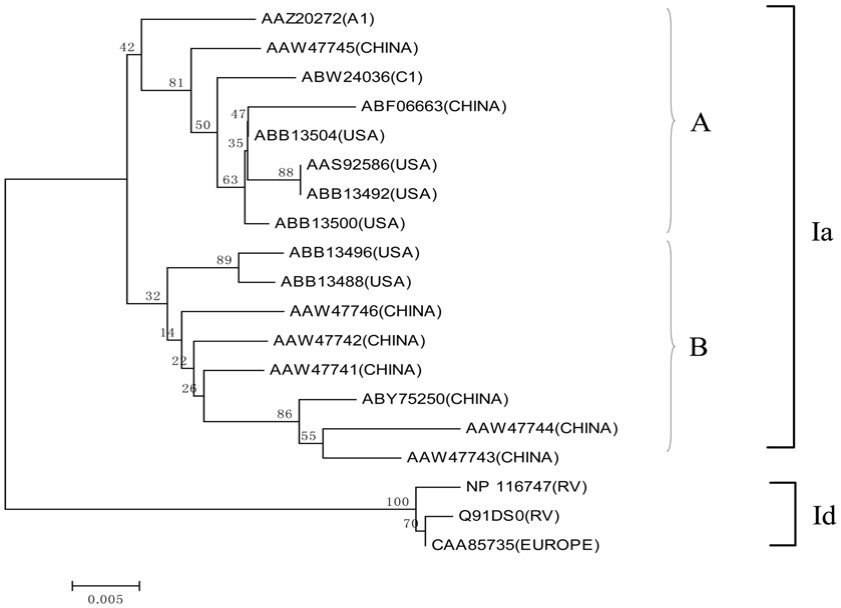
The phylogenetic tree generated by Neighbor-Joining method based on the G proteins from all SVCV isolates. Two genogroups, Ia and Id were differentiated by using the p-distance substitution model with bootstrap test of 1,000 replicates. SVCV G proteins isolated in China belong to genogroup Ia which can be further divided into Ia-A and Ia-B sub genogroups. SVCV-C1 belongs to Ia-A subgroup. Country origins of the isolates are shown in the brackets. RV represents the reference virus.

The G protein interacts with its receptor and mediates the virus entry into the host cells. It is the antigen that determines the serological properties of the SVCV [2]. Based on analysis of G genes, the isolated SVCV strains were classified into four subgroups, Ia-Id [18]. Warg et al. demonstrated that the diversity of 3843–3902 nucleotides in the genome is the key feature identifying Ia-Id genogroups [10]. Using full-length G protein sequences of Asian strains, a phylogenetic tree was constructed to determine the relationship of Asian isolates with the other isolates (Fig. 2). The Asian isolates belongs to Ia genogroup and can be further divided into Ia-A and Ia-B sub genogroups. The G protein of SVCV-C1 appeared to be closely related to the Missouri (ABB13504) and Washington isolates (ABB13500) [10].

**Figure 3:**
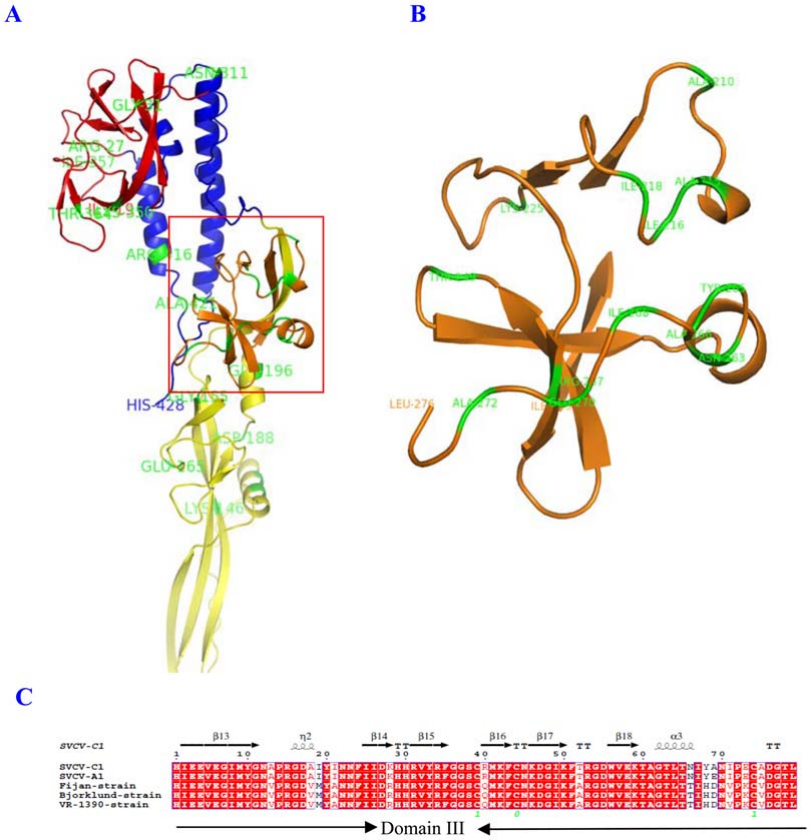
The 3D structure and alignment of SVCV G proteins (domain III). A, Four domains are colored: red (domain I: 19–35aa, 328–401aa), blue (domain II: 36–53aa, 277–327aa, 402–428aa), orange (domain III: 54–68aa, 199–276aa), yellow (domain IV: 69–198aa, yellow). Variations of amino acids between SVCV-C1 and other four strains are colored with green. B, The 3D structure of domain III in G protein showing the locations of amino acid variations. C, Multiple alignment of domain III of G protein. Protein secondary structures (α helices and β sheets) are indicated above the amino acid sequences. *GenBank accession numbers used are: SVCV-C1, EU177782; SVCV-A1, DQ097384; Fijan strain, AJ318079; Bjorklund strain, NC_002803; VR-1390 strain, U18101.

SVCV is classified as a tentative member of genus *Vesiculovirus* (VSV) in the family *Rhabdoviridae*. It is possible that like VSV, entry of the SVCV into the host cells could be mediated by the G protein. The trimers G proteins form spikes on the virus surface and induce viral endocytosis [2]. The crystal structure of VSV G protein was reported recently [29]. Using the three-dimensional structure (3D) of VSV-G protein as a template [30], we modelled the predicted 3D structure of SVCV-C1 G protein on the SWISS-MODEL website (http://swissmodel.expasy.org/workspace/) (Fig. 3). Four domains in the SVCV-C1 G protein were determined by alignment and comparison of the secondary structures with PyMOL software (DeLano Scientific, http://www.pymol.org). Domain I is composed of two segments (19–35, 328–401 aa) which lie on the top of G protein, and contain 3 antiparallel β sheets. A potential glycosylation site is located at position 336 aa in domain I. Domain II consists of three segments (36–53, 277–327, 402–428 aa), forming 3 α helices. The longest helix (279–312 aa) is likely to be involved in trimerization and determine the conformational change reversibility by the pleckstrin homology (PH) conditions. The transmembrane region is located at the C terminus of the second longest helix (402–418 aa). There are several conservative amino acids, such as I^96^, I^100^, D^87^, R^89^, W^90^, Y^91^, P^119^, A^135^, H^150^, H^180^, which might be used to maintain the stability of the domain. Domain III is composed of two segments (54–68, 199–276) and has two β sheets and two α helices that constitute a PH domain (Fig. 3B). C^236^ and C^271^ form a disulfide bridge that stabilizes β sheet structure. Domain IV (69–198 aa) contains a six-stranded β barrel near the domain III, and a three-stranded β barrel at the other end. It is stabilized by the disulfide bridge linking C^171^ and C^176^. Domain III and IV are linked by disulfide bond of C^195^ and C^241^ [29].

The variable amino acid sequences between SVCV-C1 and European reference strains are present in domain III and IV. Five amino acid substitutions occurred in domain IV. Since these substituted residues are similar, they have little impact on the domain structure. Notable differences are in the loops of domain III, AIYI–VMYA (215–218 aa), NIYANIPECA–TIHDNVPKCV (263–272 aa), resulting in changes of PH domains [31]. However, the C^271^, which participates in formation of the disulfide bridge with C^236^, is conserved (Fig. 3C). PH domain plays a role in recruiting proteins to the membrane of host cells, it is possible that the infectivity of the SVCV strains may be different [32].

### Real-time Quantitative RT-PCR to Detect SVCV

**Figure 4:**
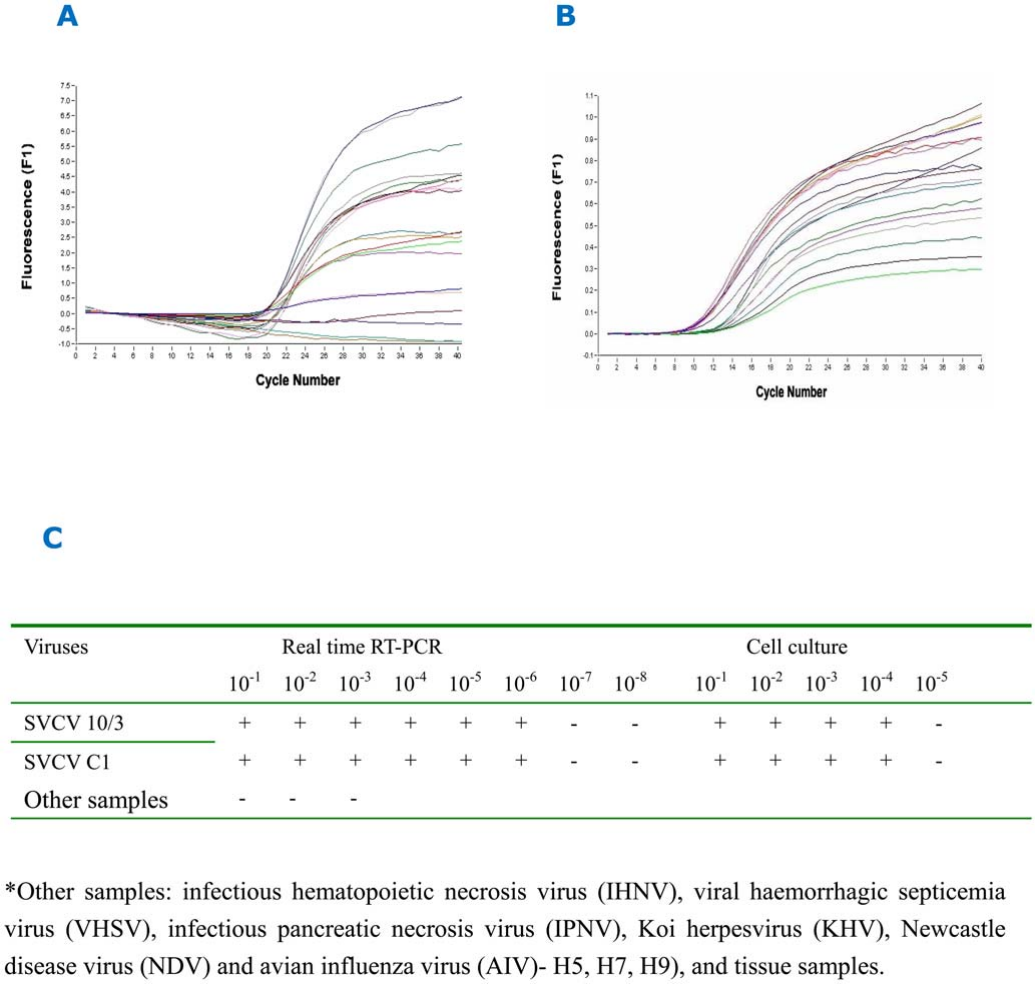
Optimization of real-time quantitative RT- PCR assay. A, optimization of primer and probe concentration. B, optimization of Mg2+ concentration. C, comparison of the sensitivity between real time RT-PCR assay and cell culture method.

Real-time quantitative RT- PCR (qPCR) assay was optimized by multiple tests (Fig. 4). The values of cycle threshold (Ct) reached a stable level at 11.55, when primers and probe were used at 0.2 µM and 0.1 µM respectively. The qPCR based approach was able to detect viruses in 10^−1^∼10^−6^ dilution of the SVCV sample whilst the standard cell culture method detected was 10^−1^∼10^−4^ 50% tissue culture infective dose (TCID_50_) of the same sample (Fig. 4C), demonstrating that the qPCR was 100 times more sensitive. The qPCR assay was also highly specific and did not amplify non-specific products from other viruses (infectious hematopoietic necrosis virus (IHNV), Viral haemorrhagic septicemia virus (VHSV), infectious pancreatic necrosis virus (IPNV), Koi herpesvirus (KHV), Newcastle disease virus (NDV) and avian influenza virus (AIV)- H5, H7, H9), and a wide range of cell and tissue samples (CO cell, EPC cell, FHM cell, eggs of sturgeon and salmon, muscle, pancreas, gill, spleen, fin, brain, kidney of ornamental carps, common carps, goldfishes, and grass goldfish).

### Epidemiological Investigation in Ornamental Fish Farms

A standard cell culture method recommended by OIE was applied for SVCV epidemiological investigation for the last five years. A large number of fish (1190∼1600) were tested each ornamental fish farm every year. In 2005, one single SVCV strain (SVCV-C1) was isolated from 242 samples and in 2006, another strain was obtained from 238 samples (Table 2). CPE developed 3 days after the cultured cells inoculated with diluted viruses from the two positive samples, with appearance of rounded cell, detachment, and netting. Complete cell detachment occurred in 4–6 days. CO cells was more susceptible to SVCV infection than EPC cells, with 10^−1^∼10^−4^ TCID_50_ detected in the CO cells. In addition, two positive samples were detected by real-time quantitative RT-PCR assay from 146 samples collected from 2005 to 2006.

**Table 2:** SVCV molecular epidemiological investigation from 2002 to 2006.

Years	Quantity of fish	Quantity of sample	Cell culture		qRT-PCR	
			positive	negative	positive	negative
2002	1600	320	0	320		
2003	1450	290	0	320		
2004	1250	250	0	250		
2005	1210	242	1	240	1	114
2006	1190	238	1	237	1	32

* The two positive simples in cell culture and qRT-PCR are same simples.

## Discussion

To date no SVCV outbreak has occurred in China. Two world events sparked our great interest in studying SVCV in China. In 1998, SVCV was isolated in goldfish and ornamental carp exported to England from Beijing. In 2002, SVC was recorded in the USA, and that the viral pathogen SVCV was suggested to be linked with imported fish, likely from China, based on gene sequence analysis [11]. The G genes of the isolated SVCV strains in the two incidents were sequenced and named as “Asian strain”. It is uncertain as to whether SVCV indeed originated from China? For this, we have developed ELISA and real-time quantitative RT-PCR methods in an attempt to isolate and identify SVCV in Beijing area since 2002. From 2002 to 2006, we tested samples from many ornamental fish farms by a cell culture method, and identified only two positive samples from over 7000 fishes located in Miyun of Beijing. The whole genome of the isolated SVCV-C1 was sequenced and analyzed. Our results indicated that there was latent SVCV in ornamental fish farms in northern China.

Interestingly, during preparation of this manuscript, a research paper describing the complete genomic sequence of SVCV-A1 strain was published and the sequence (DQ097384) was deposited in GenBank [12]. For some reason, this sequence was withdrawn from the database. On the other hand, the three SVCV reference full length sequences (Fijan strain, AJ318079; Bjorklund strain, NC_002803; VR-1390 strain, U18101) used in the comparison were simply submitted by different researchers for the same virus. In spite of this circumstance we have used the information from the SVCV-A1 and the other three SVCV strains, and even used partial genome sequences for comparison. By comparing the genomes of SVCV-C1 with other SVCV strains released in the GenBank, no essential difference was observed in the arrangement of ORF and gene junctions, but there were two differences which can be used as the molecular markers for SVCV-C1. When compared with other strains, six amino acids (KSLANA) were inserted in L protein between 376–381 aa and a 10 nucleotides were insertion in the junction region between G and L gene. It must be noted that the 10 nucleotide insertion was also found in five of the US isolates [10]. Thus, it is possible that the five SVCV isolates characterized in the United States could be introduced from China directly or via a third party.

Currently, methods used to detect SVCV include ELISA [33], [34], immunofluorescent antibody test [35], RT-PCR and so on [9], [36]. Single serotype of SVCV made it easy to establish the immunological detection methods. The OD value of the ELISA assay we established can reach as high as 1.19, without significant difference between SVCV-C1 and SVCV (10/3) reference strains. Recently, Yue et al. have reported the fluorescent RT-PCR technique for the detection of SVCV [37]. Specificity and sensitivity are crucial for this approach where the probe, primers and PCR reaction condition are the key factors affecting the results [38]. The primers and probe in our real-time quantitative RT-PCR are highly conserved and therefore, the assay can accurately detect SVCV as low as 10^−6^ TCID_50_. No other viruses, such as AIV and others, were shown to be positive, confirming the specificity of our assay.

G protein is the immune protective antigen protein for SVC, and DNA vaccination against G protein provides strong anti-virus protection [39]. Furthermore, the G gene is also used to identify the genotype of SVCV [17]. In order to compare the differences of the spatial locations due to the amino acid variation, the homology modeling of SVCV-C1 G proteins was performed by using the 3D structure of VSV G protein as a template. Two obvious differences at positions 183–187 and 232–241 were observed. They were both located in the PH domain of the 3D of SVCV-C1. PH domain is a common biological structure with a variety of functions, and plays an important role during the virus invasion of host cells [40]. The differences mainly lie in the relative changeable loops connecting β sheets, and hence, they may affect the infectivity and the T and B cells epitopes of SVCV with little change of basic structure of G proteins.

In summary, SVCV-C1 was isolated and its whole genome sequenced. The 3D structure of G protein was modeled and homology analyzed. ELISA and real-time quantitative RT-PCR assays were established and utilized to perform the epidemiological investigation. The results confirm the existence of SVCV in ornamental fish farms in northern China, which could be the origin of European and American isolates. The Chinese SVCV has some unique molecular hallmarks compared with its European and American counterparts. Based on the high infectivity of SVCV, we speculate that SVC, a typical fatal viral disease infecting cyprinid fish, may have well spread in East Asian.

## Methods

### Viruses and Cell Lines

The European reference strain of SVCV (10/3), IHNV, VHSV, IPNV, KHV, NDV and AIV- H5, H7, H9 were maintained by aquatic animal laboratory of Beijing Entry-Exit Inspection and Quarantine. The cell lines of EPC and CO were kindly provided by Professor Yuling Jiang, Shenzhen Entry-Exit Inspection and Quarantine of China.

### ELISA Assay for SVCV

SVCV (10/3) was inoculated to monolayer EPC cells grown for 24–48 hours. After absorbing for 1 hour at 20°C, the supernatant was removed, and 199 cell culture medium supplemented with 2% fetal bovine serum (FBS) was added. When the CPE reached 90%, the culture cells were frozen and thawed three times. The supernatant was harvested by centrifugation at 8,000 r/min for 1 hour. Furthermore, the viruses were collected by centrifugation at 35,000 r/min for 3 hours. The pellet was resuspended in phosphate buffered saline (PBS), and collected by 30% sucrose density gradient centrifugation at 45,000 r/min for 5 hours. The pellet of viruses was resuspended in PBS again. The sucrose was removed by centrifugation at 40,000 r/min for 3 hours and the pellet resuspended in PBS. The concentration of viruses was determined with ultraviolet spectrophotometer. Two New Zealand white rabbits were used to generate high levels of binding antibody against SVCV (10/3) [41]. In briefly, rabbit (weight 3.5 kg) was injected subcutaneously with 2.0 ml volumes of SVCV10/3 suspension (1 mg) emulsified in complete Freunds adjuvant; boost rabbit 2 weeks later with 2.0 ml volumes of SVCV10/3 (1 mg) emulsified in incomplete Freunds adjuvant; repeat booster immunization 2 weeks after the initial boost. Seven days later, 40–50 ml of venous blood were taken from each rabbit and, after centrifugation, the serum was removed and stored. A double sandwich ELISA method for the detection of SVCV was established according to the method described by Cardoso et al. [42].

### Isolation of SVCV Strain

According to the cell culturing method recommended by OIE, SVCV was isolated from the fish samples collected in 2005 (http://www.oie.int/eng/normes/fmanual/A_00021.htm). First, the tissues (brain, kidney, and spleen) of five fishes were mixed and homogenized. Subsequently, a dilution of the 1/10 organ homogenate was made with medium 199 supplemented with 2% FBS, 1,000 U/ml penicillin, 800 µg/ml streptomycin, and was buffered to pH 7.4 with sodium bicarbonate. After incubating for 2–4 hours at 15°C, the supernatant was collected by centrifugation at 7,000 r/min for 15 min. Following sterile filtration, tenfold dilution series of the supernatant ranging from 10^−1^ to 10^−3^ was inoculated to monolayer EPC or FHM cell line with 100 µL of each dilution per well (96-well microtitre plate). After absorbing for 0.5 hour at 15°C, medium 199 (supplemented with 10% FBS), 1,000 U/ml penicillin, 800 µg/ml streptomycin) was added with 200 µL per well and incubated at 20°C. The European reference isolate of SVCV (10/3) was inoculated as the positive control, and the cells without SVCV were used as the blank control. The CPE was examined daily with phase-contrast microscope. If no CPE developed in the inoculated cultures, the cells were cultured for further 7 days. If there were still no CPE observed, the test was declared negative. Further ELISA test was conducted if CPE developed.

### Infection Test by the Isolated SVCV Strain

Ten healthy fishes were equally distributed into two groups and reared in recirculating systems, and the water temperature was maintained at 18–22°C. One group of fish was infected with SVCV-C1 by oral and branchial administration and the other used as the control. After 40 days, fish were killed and tissues (gill, brain, kidney, intestine, liver, spleen, muscle, gallbladder, and pancreas) were collected. Water samples were also collected. The SVCV-C1 strain was isolated from tissue and water samples as previously described.

### The Genome Sequence

Nine pairs of primers (synthesized by Shanghai Sanggon Biological Engineering Technology & Services Co. Ltd) for the SVCV-C1 genome were derived from four released genome sequences of SVCV strains (AJ318079, NC_002803, U18101, DQ097384) and are listed (Table S1). The genomic RNA of SVCV-C1 was extracted with Trizol (Inventrogen, USA) according to the manufacturer's instructions and was desolved in DEPC-treated water. First cDNA strand was synthesized with a first-strand cDNA synthesis kit (Promega, USA) according to the instructions. The one step RT-PCR system (50 µL) is: 10×RT buffer 5 µL, MgCl_2_ 10 µL, dNTPs 5 µL, RNase Free Water 15 µL, RNase Inhibitor 1 µL, forward prime 1 µL (50 pmol), reverse primer 1 µL (50 pmol), Optimized Tag 1 µL, AMV-RTase 1 µL, template RNA 10 µL. The annealing temperatures of the reactions are listed in Table 1. The PCR products were analyzed by electrophoresis on 0.8% agarose gels, purified, and cloned into the pGEM-T easy vector (Promega, USA) for sequencing. The plasmids of positive clones were sequenced by TAKARA BIOTECHNOLOGY (DAILIAN) CO. LTD. DNASis V2.5 was used to create the contigs and assemble the genome. Putative ORFs were predicted by submitting to NCBI ORF finder. DNAMAN was applied to analyze the differences of the four genome sequences of SVCV. The 3D structure of SVCV-C1 G protein was predicted by submitting its amino acid sequence to SWISS-MODEL Workspace. The structure was analyzed with PyMOL software.

### Real-Time Quantitative RT-PCR

The complete G genes of SVCV released in GenBank were aligned using DNAman software. The primers and probe were derived from the conserved region of G genes and designed by using the Primer Express software Version 3.0 (Applied Biosystems, Foster City, CA) (Fig. S2). Both of the forward and reverse primers have 20 nucleotides, and the probe has 25 nucleotides, making its Tm is 5°C higher than the primers. Standard curve of real-time quantitative RT-PCR was constructed using the serial dilutions of the reference SVCV (10/3) G gene RNA (one copy∼10^10^ copies). Serial 10-fold dilutions of cell culture inoculated with SVCV (10/3) from 10^−1^ to 10^−10^ were divided into two parts, half was detected by real-time quantitative RT-PCR to test the sensitivity of the real-time quantitative RT-PCR for SVCV , and the other half was screened by standard cell culture (CO cell) method to determine the TCID_50_. The 50 µL optimized real-time quantitative RT-PCR mixtures contained: up primer 1 µL, low primer 1 µL, template 1 µL, 10 Taq (40U) 0.5 µL, Ready-To-Go RT-PCR Beads one pellet (Amersham, USA), water treated with DEPC 37.5 µL. The optimum system was performed at 42°C for 30 min, followed by 94°C for 3 min, then by a three-step cycle pattern consisting of 40 cycles at 94°C for 30 Sec,55°C for 30 Sec, and 72°C for 1 min. Every reaction was repeated for 3 times. Furthermore, IHNV, VHSV, IPNV, KHV, NDV, AIV (H5, H7, H9), CO cell, EPC cell, FHM cell, eggs of sturgeon and salmon, tissues (muscle, pancreas, gill, spleen, fin, brain, kidney) of ornamental carps, common carps, goldfishes, and grass goldfish were used as control templates to verify the specificity of the method.

### Epidemiological Investigation

From 2002 to 2006, over 1300 samples from 15 ornamental fish farms had been tested using the two methods established. Total 6700 individuals were examined for SVCV presence by cell culturing. Samples were selected as follow: whole alevin (body length<or = 4 cm), entire viscera including kidney and encephalon (>4 cm body length<and = 6 cm) or, for large fish, liver, kidney, spleen and encephalon. The tissue samples of 5 fishes were mixed and homogenized. The SVCV was isolated as described previously. One hundred and forty nine samples were examined by the real-time quantitative RT-PCR in this study.

## Supporting Information

Figure S1 The genomic sequence of SVCV-C1 deposited in GenBank under accession number EU177782.
(0.02 MB PDF)

Figure S2 Information of primers and probe for real-time quantitative RT-PCR for detecting SVCV strains.
(0.03 MB PDF)

Table S1 The primers for the genome sequencing of SVCV-C1.
(0.03 MB PDF)
