# Supplementary material for: Molecular Analysis of Spring Viraemia of Carp Virus in China: A Fatal Aquatic Viral Disease that Might Spread in East Asian
Source: PLoS One. 2009 Jul 22;4(7):e6337. doi: 10.1371/journal.pone.0006337 (PMC2710009; doi:10.1371/journal.pone.0006337)
Supplement: Figure S1 — The genomic sequence of SVCV-C1 deposited in GenBank under accession number EU177782. (0.02 MB PDF) [file pone.0006337.s001.pdf]

Figure S1. The genomic sequence of SVCV-C1. It was deposited in GeneBank with accession number EU177782.

```
LOCUS       EU177782            11047 bp    cRNA    linear   VRL 17-OCT-2007
DEFINITION  Spring viremia of carp virus isolate BJ0505-2, complete genome.
ACCESSION   EU177782
VERSION     EU177782.1  GI:158252063
KEYWORDS    .
SOURCE      Spring viremia of carp virus
  ORGANISM  Spring viremia of carp virus
            Viruses; ssRNA negative-strand viruses; Mononegavirales;
            Rhabdoviridae; Dimarhabdovirus supergroup; Vesiculovirus.
REFERENCE   1  (bases 1 to 11047)
  AUTHORS   Zhang,N.Z., Jiang,Y.N., Xu,J.M., Zhang,T. and Xia,C.
  TITLE     Spring Viremia of Carp isolated from Beijing, complete genome
  JOURNAL   Unpublished
REFERENCE   2  (bases 1 to 11047)
  AUTHORS   Zhang,N.Z., Jiang,Y.N., Xu,J.M., Zhang,T. and Xia,C.
  TITLE     Direct Submission
  JOURNAL   Submitted (25-SEP-2007) College of Animal Medicine, China
            Agricultural University, YuanMingYuan XiLu 2, HaiDian District,
            Beijing 100094, China
FEATURES             Location/Qualifiers
     source          1..11047
                     /organism="Spring viremia of carp virus"
                     /mol_type="viral cRNA"
                     /isolate="BJ0505-2"
                     /db_xref="taxon:11275"
                     /country="China: Beijing"
     misc_feature    1..59
                     /note="putative leader region trailer region"
     gene            60..1394
                     /gene="N"
     mRNA            60..1394
                     /gene="N"
                     /product="nucleoprotein"
     CDS             70..1326
                     /gene="N"
                     /codon_start=1
                     /product="nucleoprotein"
                     /protein_id="ABW24033.1"
                     /db_xref="GI:158252064"
                     /translation="MSVIRIKTNATVAAVLPANEDQADYPSTFFEGGNEIRLYVNRGE
                     KLDVLRQYVYMGLVEKNCKIQHVNAYLYAVLKGERELLEADWDSFGHKIGTQGDKIGP
                     FNLVRVEDIPDGLPDGKLNAEVSAEDDAWLPLFLLGLYRVGRASETAYRTLLMESLIK
                     QCKAIKSDWVSPVTATHKYFDVWGNDGNYLKIVACVDMFYDHFKKSIKATFRWGTIVS
                     RFKDCAALATLGHVVKITGLTIEEVFTWVLQTEVADELVKMMKPGQEIDKSTSYMPYL
                     IDMGISAKSPYSTIKNPSFHFWGQLVAALCRSKRALNARQPDEIDSMSISNASLLMAY
                     ALGSSPDIEQQFSTGDTYRKPPKEASYLVSEEPKNRSVVEWIAWYSDVDNKPTDDMLM
                     MAKRVAGTISGPRDDSVGKWIKQTYG"
     gene            1397..2363
                     /gene="P"
     mRNA            1397..2363
                     /gene="P"
                     /product="phosphoprotein"
     CDS             1407..2336
                     /gene="P"
                     /codon_start=1
                     /product="phosphoprotein"
                     /protein_id="ABW24034.1"
                     /db_xref="GI:158252065"
                     /translation="MSLHSKLSESLKAYANLEKTVKEIEEQVSIMEEPVPKTVKYVTF
                     EEDLSEGDWESDSGDDDEDSIDESLIPDYLRESSSITVDEDEEDQKADEEEYLPTVSW
                     EEESTGIDLGFGPGIVMPSVSDHEGGTYVRYNGLGNIDPNYKNLISKMMRSLIGQIGN
                     KYGYDIDLFDYQGDFLEVFLPHKPSKEDVRPGIRTEKKNEESPGKQVSKPEKKEKTIQ
                     KTGDECGRFPMDKEAKRREPEGLWEIMKVLSVQFDPWKEDEPPLNMTIRDLFISESEF
                     RLHCNHSQTEREMALVGIRLRRLYNKLYQKYRL"
     gene            2366..3081
                     /gene="M"
     mRNA            2366..3081
                     /gene="M"
```

/product="matrix protein"
CDS 2376..3047
/gene="M"
/codon_start=1
/product="matrix protein"
/protein_id="ABW24035.1"
/db_xref="GI:158252066"
/translation="MSTLRKLFGIKKSKGTPPTYEETLATAPVLMDTHDTHSHSLQWM
RYHVELDIQLDTPLKTMSDLLGLLKNWDVDYKGSRNKRRFYRLIMFRCALELKHVSGT
YSVDGSALYSNKVQGSCYVPHRFGQMPPFKREIEVFRYPVHQHGYNGVVDLRMSICDL
NGEKTGLNLLKECQVAHPNHFQKYLEEVGLEAACSATGEWILDWTFPMPVDVVPRVPS
LFMGD"
gene 3084..4681
/gene="G"
mRNA 3084..4681
/gene="G"
/product="glycoprotein"
CDS 3094..4623
/gene="G"
/codon_start=1
/product="glycoprotein"
/protein_id="ABW24036.1"
/db_xref="GI:158252067"
/translation="MSIISYIAFLLLIDSTLGIPIFVPSGRNISGQPVIQPFDYQCPI
HGNLPNTMGLSATKLTIKSPSVFSTDKVSGWICHAAEWKTTCDYRWYGPQYITHSIHP
ISPTIDECKRIISRIASGTDEDLGFPPQSCGWASVTTVSNTNYKVVPHSVHLGPYGGH
WIDHEFNGGECREKVCEMKGNHSIWITDETVQHECEKHIEEVEGIMYGNAPRGDAIYI
NNFIIDKHHRVYRFGGSCRMKFCNKDGIKFTRGDWVEKTAGTLTNIYANIPECADGTL
VSGHRPGLDLIDTVFNLENVVEYTLCEGTKRKINNQEKLTSVDLSYLAPRIGGFGSVF
RVRNGTLERGSTTYIKIEVEGPIVDSLNGTDPRTNASRVFWDDWELDGNIYQGFNGVY
KGKDGKIHIPLNMIESGIIDDELRHAFQADIIPHPHYDDDEIREDDIFFDNTGENGNP
VDAVVEWVSGWGTSLKFFGTTLVALILIFLLIRCCVACTYLMKKSKRPATESHEMRSF
V"
gene 4686..11028
/gene="L"
mRNA 4686..11028
/gene="L"
/product="L protein"
CDS 4696..11001
/gene="L"
/codon_start=1
/product="L protein"
/protein_id="ABW24037.1"
/db_xref="GI:158252068"
/translation="MFEWESQDTPSGLPDEESYFPTSKLSVEERMHYLNNVDYNLNSP
LISDDIEYLTLRHFGRAIPSLWKIKNWEIPLEMLKGVGIIKTWDQIHPWMGRWFDSEH
NCPQGESFLRTVQAESELTSEIPVTFIKGWIGKEIKFPVKRGHHAVHLLMQKVLDLHK
LTLLINSVDSSETEKLCESFGLHPKQSKFETHSLGTVRYCPGWIFIEKAEILLDRNFL
LMMKDTLIGRLQTLLSMLGNCEMEIEQICTHTETMLSLYSYGDQIIEKSGNNGYSKIK
LLEPICNLRLSELAHKYRPLVPDFPHFQEHVETSVREEDTPEGLLSAILSLVNNTEDI
QLILTIYGSFRHWGHPFISYFEGLQKLHDQVTLPKQIDREYAKSLANAAALASDLAYT
VLQRKFSEEKKWYVDSITLSQKHPLKEHVDNGTWPTAAQIQDFGDRWHLLPLTKCFEV
PDLLDPSVIYSDKSHSMNRKEVIDHVISTPNKPIPSKKVLETMINNPATDWPTFLKAV
DEEGLPRDSLIIGLKGKERELKIAGRFFSLMSWQLREYFVITEYLIKTHYVPLFKGLT
MADDLTSVVKKMLDNTNGQGLDDYSSICIANHIDYEKWNNHQRKESNGPVFRVMGQFL
GYPRLFERTHEFFESSLIYYNGRPDLMDVRGDSLVNTTDRIVCWEGQAGGLEGLRQKG
WSVLNLLVINRESSIRNTVVKVLAQGGNQVICTQYKTKNYKNEEELRMLLTAMVENNQ
TIMNGIIAGTGKLGLIINNDETMQSADYLNYGKVPVFRGILRGLETKRWSRVTCITND
QIPTLAGVMSSVSTNALTVAHFAASPINAILQYHYFANFCLMMIAMHNPAIRSSMYTK
MFRKCHIMSREFKAVTLYLDPSLGGVCGISLARFLIRSFPDPVTEGLAFWKMIHHNCQ
SDWLKALSKRCGNPKLARFRPEHIPKIIEDPATLNISMGMSASNLLKTEVKGHLIRTA
DTIQNQIIREAAEYLGQEEESLNEFLWDIEPFFPRFLSEFRSSTFVGVTDSLIGLFQN
SKTIRGLFKSYYKRELDRLVVKSELSSLEHLGSYRKETPDSIWECSSTQADLLREKSW
GRSVIGMTVPHPLEMFGKGHQKELECTPCQTSGLTYISSYCPKGINNWYSTVGSLAAY
LGSKTSETTSILQPWEKDSKIPIIKRATKLRDSISWFVPPDSKLAKSIQQNLKALTGE
DWEEDIQGFKRTGSALHRFTTSRVSNGGFSAQSPAKLTRIMTTTDTMRDLGDQNYDFM
FQAGILYSQMTTGELRENSTNSTATHYHITCKSCLREIQEPMLESRIIYNPPSSSRVI
KSWIPNATEIMEESKPVKLREVDWDPLTRYEKSYHIGRCQGFLYGDLTYQKTGRSEES
SIFPLSIQYKVEGSGFMRGFCDGTIRASAVQALHRRVSSIVSTADVIYGGALYLTNQV
GDSPPFQNLCRSGPLREELERIPHKMTSSYPTSNSDMGYLIRNYLKRSLKQLSRGRYE
TRDGPIWVFSDVRTKKFLGPFSLSTDALNCLYKNKLSKKDKNAVRNLSQLSSRMRSGD
LSDEEIGKIEARFSFTPAEMRHACKFTIGKTQVPIVMSEWGQEAYGNITMYPVFYSTI
KTEKPDWTFSRLQNPTISGLRISQQATGAHYKLRSLLKGMKIHYQDAIGCGDGSGGLS
SCLLRENKHCRVIFNSLLELTGNTLRGSTPDPPSAINGIPQVRDRCVNLNSVWEHPSD
LSHPDTWKYFGELKAQFNMDIDLIVMDMEVQDIDISRRIEQNLRDHVHSLLSRHGTVI
YKTYMTILSENERSVLDIVGVLFEDVQLCQTQYSSSQTSEVYCVTRRLRQKVGSQHVE
WQSLVRQGINSKVYCNLPLDKEFERALNLYQIDTLVGVPRELIPNLAVELETLLEIGG

```
                     LSGGILGKLVLNIEEGKLGFTMALIVSCILISESAICTTRLSNKREVPSSGACQRMAV
                     CLIGAAILLSVHHRSIENHKGAIRMLRHSVPIRISSKLRKDGKLQSRWSSISREGLAK
                     DVRLNSNMAGVGAWIRVWSRMKDRERRWEAREADSWLKTNNKGLSMEHVRRNTGVLDI
                     LHGTGDRLDRSVPTVSSAPRDSGTWVE"
ORIGIN
        1 acgaagacaa ataaaccatt gataacatta catggtttat ttgtcttccg aaaaatggta
       61 acagacatca tgagtgtcat tcggatcaaa acaaatgcta cagttgctgc cgtgcttccg
      121 gctaacgaag atcaggccga ttatccttcc acttttttg aaggggggaa tgagattaga
      181 ttgtatgtta acaggggggа gaaattggat gttttaaggc aatatgtcta tatgggactg
      241 gtggagaaaa actgtaagat acagcatgtg aatgcttatc tatatgctgt gctgaaggga
      301 gaaagagagc tgctagaagc ggattgggat agctttgggc acaagattgg gactcagggg
      361 gataagatcg ggccctttaa cctggtgcga gtagaagaca tccccgacgg gttaccagat
      421 gggaaactga acgcagaggt gagtgctgag gatgatgcat ggctgcctct cttcttgctg
      481 ggtctctaca gagtgggaag agcaagtgag actgcatacc ggactctgct gatggagtcc
      541 ctgataaaac agtgtaaggc aataaaatcc gactgggtat ctcctgtaac ggcaactcac
      601 aaatatttcg atgtctgggg caatgatggg aattacctga agattgtggc ctgtgtggac
      661 atgttttacg accattttaa aaagagcatt aaagcaacat ttcggtgggg aacgattgta
      721 tcacggttca aagactgtgc tgcactcgcc accctgggac atgttgttaa aatcaccggt
      781 ttgaccattg aagaggtgtt cacatgggta ctgcagactg aagtcgcgga tgagttagtc
      841 aaaatgatga agcctggaca ggagatagat aaaagcacgt cttacatgcc gtacctgatt
      901 gatatgggaa tctctgccaa atcaccatac tcaacaataa agaatccgtc ttttcatttc
      961 tgggggcagc ttgttgctgc attgtgccgc tccaagagag cactgaacgc aagacagcct
     1021 gatgagattg actcaatgtc tatctcaaat gcaagcctgc tgatggctta cgcattaggc
     1081 agcagccctg acattgagca gcaattcagt acaggagaca catacagaaa accgccgaaa
     1141 gaggcttcgt acctggtgag tgaggaaccg aaaaaccgat ctgtcgttga atggattgca
     1201 tggtattctg acgtggacaa caaacctacg gatgacatgc tcatgatggc aaaacgagta
     1261 gcagggacta tctctgggcc tcgcgatgac tcagttggca aatggataaa acaaacctat
     1321 ggataaggat aatcacatca cactgcaatg attttaggta ataagagaag tagtagttga
     1381 tagtatgaaa aaaactaaca gagatcatgt ctctacattc gaaattgtca gaaagtctaa
     1441 aagcttatgc taatttggag aagacggtta aagaaataga agaacaggta tcgattatgg
     1501 aagagcccgt tccaaagaca gtaaaatatg ttacctttga ggaggacttg tctgaggggg
     1561 actgggaatc agattcggga gatgatgatg aggattcaat cgatgaatct ttgatccctg
     1621 attacctcag agaaagtagc agcatcacag tggatgaaga tgaagaagat cagaaagcag
     1681 atgaagaaga atatcttccg acagtcagtt gggaagaaga atctacagga atagatctag
     1741 ggtttggacc tgggatagtg atgccgtctg tgtcggacca tgagggaggt acatatgttc
     1801 gttataacgg cctcggtaac atagacccaa attataagaa cttgatttct aaaatgatga
     1861 gaagcctgat tgggcaaatt ggaaacaaat atggatatga cattgatcta tttgattatc
     1921 agggagattt cctggaggtg ttcttacccc acaagccaag caaagaggat gtccgacctg
     1981 gcatacgtac agagaaaaag aatgaggaaa gccccggtaa gcaggtctcc aaacctgaga
     2041 aaaaggaaaa aaccatccag aagacggggg atgagtgcgg aagatttcct atggataaag
     2101 aggccaagag aagagaacca gagggattat gggagatcat gaaggtcttg tccgtccagt
     2161 ttgatccttg gaaagaagat gagcctccac tgaacatgac catccgagat ttattcataa
     2221 gcgaatctga gtttcgtctg cactgtaatc acagccaaac agagcgagaa atggccttgg
     2281 ttgggatcag actgaggaga ttgtacaata agttgtatca aaaatatagg ttgtaagggt
     2341 gttaaatttg gatatgaaaa aaactaacag acatcatgtc tactctaaga aagctctttg
     2401 gaatcaagaa gtcaaaaggt actcctccca cttacgagga gacactggcg actgcaccag
     2461 tattaatgga tactcatgat actcattccc actcactgca gtggatgagg taccatgttg
     2521 aattggacat acaattggac acgcccttga aaacgatgtc agaccttctc ggactcttaa
     2581 aaaattggga tgtagattac aaaggttcta ggaacaagcg tagattttac agattgatca
     2641 tgttccgttg tgcgttagaa ctcaagcatg tgtcgggaac atactctgtt gacgggtcgg
     2701 ccttgtactc caacaaggtg caaggaagtt gttatgtacc tcatcgattt ggtcaaatgc
     2761 ctcctttcaa gagagagatc gaggtcttta gatacccagt acaccaacat ggatacaacg
     2821 gggtagtaga tctaagaatg tcgatctgtg atctaaatgg agagaagaca ggcctcaacc
     2881 tgttgaaaga gtgtcaggtg gctcacccca accatttcca aaaatatcta gaggaggtcg
     2941 ggctggaggc agcctgttcg gccacaggag agtggattct tgattggaca tttcctatgc
     3001 cagtagacgt ggtgccccgt gttccttccc tgttcatggg agattaagtt gagatcaata
     3061 ttcgctgaga tatgaaaaaa actaacagac atcatgtcta tcatcagcta catcgcattc
     3121 cttttgctaa ttgattccac attgggaatc cccatatttg ttccatccgg gcggaatata
     3181 tcagggcaac ctgtaattca gccatttgat tatcaatgtc caatacacgg aaatctacct
     3241 aacacaatgg gattgagtgc caccaaattg acaataaaat ctccatctgt cttcagtaca
     3301 gataaagttt ctggatggat ctgccatgca gctgaatgga aaacaacttg tgattacaga
     3361 tggtacggac cccaatatat aacccacagt attcatccaa tcagtcctac catagatgaa
     3421 tgcaagagaa tcatttcaag gattgcatca ggaactgatg aagatctggg gtttcccccct
     3481 caaagttgcg gatgggcatc tgtcacaaca gtgtcaaata ctaattacaa ggtagtaccc
     3541 cattccgttc atttggggcc gtacggagga cactggatcg atcatgaatt caatgggggc
     3601 gaatgcagag aaaaagtgtg tgaaatgaaa gggaaccact ctatttggat cacagatgag
     3661 accgtgcagc atgaatgtga aaagcacata gaggaagttg aaggaattat gtacgggaat
     3721 gctccgagag gggatgcaat atatattaac aactttatta tagataaaca tcatagagta
     3781 tacagattcg gggggtcttg tcgaatgaaa ttctgtaata aagatggtat aaaattcaca
     3841 agaggagact gggtagaaaa aacagctgga acattgacga atatttatgc aaatatacct
     3901 gaatgtgctg atggaacgtt ggtatctggt caccgacctg gattagactt gattgacaca
     3961 gtcttcaatt tggaaaatgt ggtagaatat actttgtgtg aagggactaa aagaaaaatc
     4021 aataaccaag aaaagttgac gtcagtggat ttgagttatt tggccccaag aattggaggg
     4081 tttggatcag tattcagagt gagaaacgga acattagaga gagggagcac tacttatatc
     4141 aagatagaag tagagggacc tattgtcgac tcgttgaatg gaacagatcc gagaaccaac
     4201 gcctcaagag tattttggga cgactgggag ttagatggca atatatatca gggctttaat
     4261 ggtgtatata aagggaaaga tgggaagatc catattccct tgaatatgat agaatcagga
```

4321 atcatagatg atgaacttcg acatgctttc caagccgata ttatccctca tcctcattat
4381 gacgacgatg aaatccgaga ggacgatata ttcttcgata atactggaga aaatggaaat
4441 cccgtggatg cagtggtaga atgggtcagt gggtggggaa ctagtctaaa attctttggc
4501 acgactctgg tcgccctgat tttgatcttt ctgctcatca ggtgctgtgt tgcttgcact
4561 tatttgatga agaagagtaa acggcctgca acagaatcac acgaaatgcg gtccttcgtt
4621 tgagagatag ccaattttaa gcaaagacca agatattatc ttaataggtg tatgaaaaaa
4681 actataacag acatcatgtt tgagtgggaa agtcaggata ctccatctgg attgccagat
4741 gaggagtcct acttcccaac ttcaaaattg agtgtcgaag aacgcatgca ctatttaaat
4801 aacgtagatt acaatttgaa ttcaccttta atttcagacg acattgaata tctcacattg
4861 aggcattttg ggagagccat accatcgtta tggaaaatta agaattggga aatacccttta
4921 gaaatgttga agggagtcgg catcatcaag acctgggatc agatacatcc atggatggga
4981 aggtggttcg actcagaaca caattgtcca caaggggagt cattcttacg gacagtacaa
5041 gcagagagtg aactcacctc agaaatacca gtgaccttca ttaaaggctg gattgggaaa
5101 gaaatcaagt ttccggtaaa gagaggacat catgcggtgc atttactcat gcagaaagtc
5161 ctagatctgc acaaattgac tctgttgatt aattcagtag attccagtga gacagaaaaa
5221 ctgtgtgagt cttttggatt gcacccaaag caatcgaaat ttgagacaca ttctctggga
5281 accgtccgat actgtcctgg atggatcttc atagaaaaag ctgagatatt attagatcgc
5341 aatttcttgc tcatgatgaa agacaccttg attggacgac tacaaacatt gttatcaatg
5401 ttgggcaact gtgagatgga aatagaacaa atttgcaccc atacggagac tatgctgtcc
5461 ttatattcat atggagatca gatcatcgag aaatcaggga ataatggata cagtaagatc
5521 aaactcttag agccaatttg caacttgcgc ttgtcagaac tcgctcacaa ataccgtccc
5581 ctggtccctg acttccccca ttttcaagag cacgtggaaa catctgtacg agaagaagat
5641 accccagaag gactcctgtc cgctattcta tcactagtga acaatactga agatatacag
5701 ctgatattga ccatctatgg atcttttagg cattggggtc atccattcat atcttacttt
5761 gaggggctgc aaaagctcca cgaccaggtg acactgccta aacaaataga tagagagtat
5821 gctaaatcac tcgctaatgc tgcagcatta gcgagtgatt tagcatacac agtgttacag
5881 agaaaatttt cagaagagaa aaaatggtat gttgactcca tcactctttc ccaaaaacat
5941 cctctcaaag agcacgtgga taatggaaca tggcctactg cagcccaaat tcaagatttt
6001 ggcgatagat ggcacttgtt gcccctgacg aaatgcttcg aagttccaga cctgctagac
6061 ccgtctgtga tttactcaga taaaagccat tcgatgaaca gaaaggaagt gattgaccac
6121 gtgatatcca cacccaataa accgatcccg agcaagaagg ttttagagac catgataaac
6181 aaccctgcaa cagactggcc aacattcctt aaggcagtag atgaagaagg attacccaga
6241 gatagtctca taattggatt aaaaggaaaa gagcgagagc ttaagattgc tgggagattc
6301 ttctccctga tgtcatggca attgagagag tattttgtaa ttacagagta tctgatcaag
6361 acacattatg ttccgctctt caagggatta actatggctg atgatctcac ctcggtggta
6421 aagaagatgt tggataacac caatgggcag ggcttggatg actactcatc catctgcatt
6481 gcgaaccaca ttgattatga aaaatggaac aatcatcaac gaaaagaatc caatggccct
6541 gttttcagag tgatgggtca gttcttggga taccctagac tgtttgaaag gacacatgaa
6601 ttctttgagt cgagtctcat ctactacaac gggaggccag acctgatgga tgtgaggggg
6661 gactctttag tgaatacaac agacaggata gtttgctggg aagggcaagc gggcggatta
6721 gaagggcttc gccaaaaagg atggagtgta ctcaatctgc tggtcatcaa tagagaatca
6781 tctatcagaa acacagttgt aaaagtgttg gctcaggggg gcaatcaggt gatctgcacg
6841 cagtataaaa ccaaaaatta caaaaatgag gaagagttaa gaatgcttct gacagcgatg
6901 gtagagaaca atcagacgat catgaatggg atcatagcag ggacaggaaa actaggatta
6961 atcatcaaca atgatgagac tatgcaatcg gcggattact taaattatgg taaagtgcct
7021 gtttttcgag gaatattaag agggttggaa accaaacgat ggtccagagt cacctgcatc
7081 accaatgatc agattccaac tcttgcgggt gtaatgtctt ctgtttctac caatgctctt
7141 accgttgccc actttgcagc cagtccaatc aatgcgatat tgcaatatca ttactttgca
7201 aatttttgcc ttatgatgat tgcaatgcac aaccctgcca tccggagtag tatgtacaca
7261 aagatgttca gaaaatgtca catcatgtcc agagaattca aagctgtgac actgtatctg
7321 gatccttcat taggaggagt ctgtggcatc tccttggcta gattcttaat tagatctttt
7381 ccagatccag tgacagaggg acttgctttt tggaaaatga tacatcacaa ttgtcaatcc
7441 gattggttga aagctctatc aaaacgctgt ggaaatccta agctggcgag attcagacca
7501 gaacacatac ctaaaatcat cgaagatccg gcgaccttga atatttcaat gggaatgagt
7561 gcttctaatt tgttgaagac agaggtaaaa ggacatctga ttagaacggc agacacaatt
7621 caaaaccaaa tcatccggga ggcagcagag tatctgggac aagaggaaga atctctgaat
7681 gagttcttat gggatattga acctttcttc ccacgattct tgagtgaatt cagaagcagc
7741 acctttgttg gagttactga ctctctcatt ggattgtttc agaattcaaa aacaatacga
7801 ggattgttca agtcctacta caagagagaa ttagaccggt tggtcgtcaa gagcgaactg
7861 tcatcattag aacatcttgg ttcataccgg aaagagactc ctgattccat ctgggaatgc
7921 tctagcactc aagcagattt gcttcgagaa aagagttggg gaagatcagt aataggaatg
7981 acggtgcctc atccgttaga gatgtttggg aaaggtcatc aaaaagaact agagtgtaca
8041 ccttgtcaaa catctggact gacatatata tcctcctatt gtcccaaagg gataaacaat
8101 tggtatagca cagtgggatc actggccgct tatctgggct caaaaacgtc tgaaaccaca
8161 tccatcctgc aaccttggga aaaagatagc aaaataccta tcatcaaaag agcaacaaaa
8221 ttgagagaca gtatctcctg gtttgttccg cctgattcaa aattggccaa aagcatacaa
8281 caaaatctta aggcattgac tggagaagat tgggaagagg atatccaggg atttaagagg
8341 accggttcgg cgttgcaccg attcacaact tcaagagtca gcaacggtgg atttagtgcc
8401 caaagtcctg ccaaattgac cagaatcatg accaccacag acacaatgag agacctggga
8461 gatcaaaatt acgacttcat gtttcaagca ggcatcttat atagtcaaat gacaactgga
8521 gaattgagag agaattcgac gaattctaca gctacacact atcatatcac ttgcaagtct
8581 tgccttcgag agattcagga gcccatgtta gagtcacgaa taatctacaa ccctccatct
8641 tccagcagag tgatcaagag ctggataccc aatgcgacag aaataatgga agaatcaaaa
8701 ccagtcaagc tgagagaggt agattgggat ccgctaacca gatatgaaaa atcctaccat
8761 atcggtagat gccaagggtt cctgtatggg gatctcactt atcagaagac aggaagatca
8821 gaagagagtt ccatttttcc actcagcata caatacaaag ttgaaggaag tggatttatg
8881 cgaggatttt gtgatgggac cataagggct agtgccgtcc aggcgttgca tagaagggtg

```
 8941 tcttccattg tgtcaaccgc tgatgtgata tatggaggag cactgtatct aaccaatcag
 9001 gtaggagaca gtcctccttt ccagaacttg tgtcgatctg gtcctctgcg ggaagaattg
 9061 gagagaattc cacacaagat gacgagttcc tatcctactt caaattcaga tatggggtac
 9121 ctgatcagaa attatctgaa aagatctttg aaacaactaa gtaggggccg atatgagact
 9181 agagatggac ctatatgggt cttctctgac gtacggacga agaaattcct cgggccattc
 9241 agcttgtcaa cagatgcact aaactgtctt tacaagaaca agctgtcaaa gaaagataag
 9301 aacgcagtga ggaatctgag tcaattgtct agtagaatga gatcaggaga cttgtcagat
 9361 gaagagattg ggaagataga agcacgattt tctttcactc ctgctgaaat gcggcatgct
 9421 tgcaagttca caatcgggaa gacgcaagtt cccattgtga tgtctgaatg gggccaagaa
 9481 gcttacggga acataactat gtatcctgtt ttttactcta ccataaaaac agagaagcct
 9541 gattggacct tcagtcgatt gcagaatccg accatatctg gacttcggat aagccaacaa
 9601 gcaactggag cgcattacaa attgaggagc cttctcaagg gaatgaaaat tcactaccaa
 9661 gatgcgatcg gatgtggaga tggttcagga gggctctcca gttgtttgtt acgagaaaat
 9721 aaacactgtc gtgtcatctt taacagtctt ttggagctga caggaaacac tctgagaggc
 9781 tccactccag acccacccag tgcaattaac ggaatccctc aggtcagaga cagatgtgtc
 9841 aacttaaaca gtgtgtggga acaccccttct gatctcagtc acccagatac atggaaatac
 9901 ttcggtgaat tgaaggcaca gtttaacatg gatattgacc ttatagtcat ggatatggaa
 9961 gttcaagata ttgacatcag ccgaagaatt gaacagaatt tgagggacca cgtgcattcg
10021 ctcttatcta gacacgggac cgtcatatat aagacttaca tgaccatcct ttcagaaaat
10081 gagagatcag tacttgacat tgtcggagtc ttgtttgagg atgttcaact ctgtcaaact
10141 caatatagta gctcacagac ttcagaagtg tactgtgtta cgagacgatt aagacaaaaa
10201 gtaggcagtc agcatgtaga gtggcagagt ctagtgagac aaggaatcaa ctctaaagtg
10261 tattgtaatt tgcctttaga caaagaattc gagagagctt tgaatctata ccaaattgac
10321 acactggtcg gagtacccag agaactgatc ccaaatctgg cggtagaact ggaaaccctc
10381 ctagagatag gaggattgtc agggggaata ctgggtaaat tagtcttgaa tatcgaagaa
10441 ggaaaattgg gattcacaat ggcgttgatt gtgtcttgca ttttgatcag cgaatctgcc
10501 atatgcacta ctagactatc aaataaaagg gaagtccctt cctcgggagc ttgtcagcgg
10561 atggcagtct gtttgattgg agcggcaatt ctcctgtcag tacaccacag atcaatagaa
10621 aatcacaagg gggccattcg aatgctcaga cacagcgtgc caattcgaat ttctagtaaa
10681 ctgaggaaag acgggaaatt gcagtccaga tggtcgagca tcagtcgaga aggattggca
10741 aaagatgtac gactgaactc caatatggct ggagtagggg cttggataag agtctggtct
10801 aggatgaaag accgggaaag aaggtgggaa gctagagaag cggactcatg gctgaagaca
10861 aacaacaagg ggttatccat ggaacatgta cgaagaaata caggagtttt ggatatcctc
10921 catggaactg gagatcgatt agatcgttcg gtaccaacag tgtcttctgc tcctagagac
10981 tctgggacat gggtagaata gaagggggat tgggtagtat gaaaaaaact ggatttgtag
11041 tcttcgt
```
