# Supplementary material for: Molecular Analysis of Spring Viraemia of Carp Virus in China: A Fatal Aquatic Viral Disease that Might Spread in East Asian
Source: PLoS One. 2009 Jul 22;4(7):e6337. doi: 10.1371/journal.pone.0006337 (PMC2710009; doi:10.1371/journal.pone.0006337)
Supplement: Figure S2 — Information of primers and probe for real-time quantitative RT-PCR for detecting SVCV strains. (0.03 MB PDF) [file pone.0006337.s002.pdf]

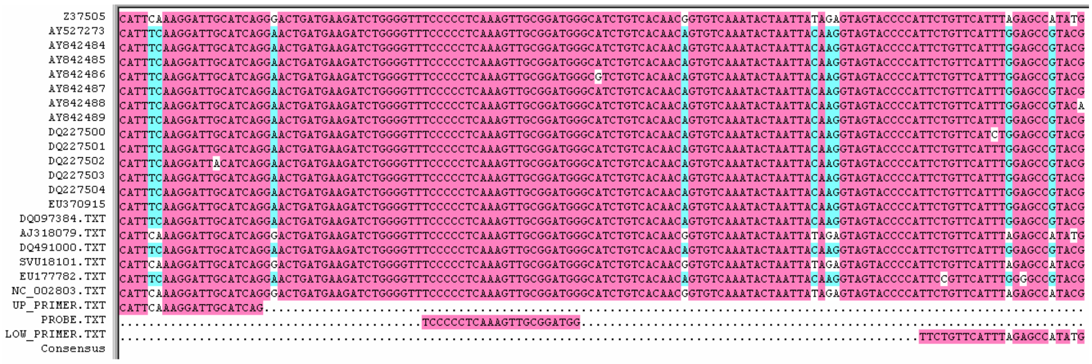

Figure S2. The design of primers and probe for real-time quantitative RT- PCR in detection of the SVCV strains.
