# Supplementary material for: Molecular Analysis of Spring Viraemia of Carp Virus in China: A Fatal Aquatic Viral Disease that Might Spread in East Asian
Source: PLoS One. 2009 Jul 22;4(7):e6337. doi: 10.1371/journal.pone.0006337 (PMC2710009; doi:10.1371/journal.pone.0006337)
Supplement: Table S1 — The primers for the genome sequencing of SVCV-C1. (0.03 MB PDF) [file pone.0006337.s003.pdf]

Table S1. The primers for the genome sequencing of SVCV-C1

Clone	Primer name	Position	Sense	Sequence(5'-3')	Annealing temperature	PCR production
**N**	NP1	3'-end	Antigenomic	ACGAAGACAAATAAACCATTGATAACAT	58℃	1439 bp
	NP2	In P gene	Genomic	TCGATACCTGTTCTTCTATTTC	58℃	
**P**	PP1	In N gene	Antigenomic	GGCAAATGGATAAAACAAACCTATG	58℃	1139 bp
	PP2	In M gene	Genomic	TAAGTGGGAGGAGTACCTTTTG	58℃	
**M**	MP1	In P gene	Antigenomic	CAGAGCGAGAAATGGCCTTGGTTGG	58℃	863 bp
	MP2	In G gene	Genomic	AGGAATGCGATGTAGCTGATGATAG	58℃	
**G**	GP1	In M gene	Antigenomic	GTTCCTTCCCTGTTCATGGGAG	58℃	1713 bp
	GP2	In L gene	Genomic	AATCCAGACGGAGTATCTTGAC	58℃	
**L**						
L1	L1P1	In G gene	Antigenomic	CAACAGAATCACACGAAATGCGG	58℃	1236 bp
	L1P2	In L gene	Genomic	TGCCAAATCACTCGCTAATGCTG	58℃	
L2	L2P1	In L gene	Antigenomic	CTGATATTGACCATCTATGGATC	58℃	1440 bp
	L2P2	In L gene	Genomic	AAGAGCATTGGTAGAAACAGAAG	58℃	
L3	L3P1	In L gene	Antigenomic	CACCAATGATCAGATTCCAACTC	58℃	1376 bp
	L3P2	In L gene	Genomic	GGTCTCTCATTGTGTCTGTGGTG	58℃	
L4	L4P1	In L gene	Antigenomic	AAGGCATTGACTGGAGAAGATTG	58℃	1487 bp
	L4P2	In L gene	Genomic	CTCAGAGTGTTTCCTGTCAGCTC	58℃	
L5	L5P1	In L gene	Antigenomic	GATCGGATGTGGAGACGGTTCAG	58℃	1392 bp
	L5P2	5'-end	Genomic	ACGAAGACTACAAATCCAGTTTTTTTC	58℃	
